# Molecular and structural insights into an asymmetric proteolytic complex (ClpP1P2) from *Mycobacterium smegmatis*

**DOI:** 10.1038/s41598-019-53736-8

**Published:** 2019-12-02

**Authors:** Jyotsna Nagpal, Jason J. Paxman, Jessica E. Zammit, Adam A. Thomas, Kaye N. Truscott, Begoña Heras, David A. Dougan

**Affiliations:** Department of Biochemistry and Genetics, La Trobe Institute for Molecular Science, La Trobe University, Melbourne, 3086 Australia

**Keywords:** Proteases, Structural biology, X-ray crystallography

## Abstract

The ClpP protease is found in all kingdoms of life, from bacteria to humans. In general, this protease forms a homo-oligomeric complex composed of 14 identical subunits, which associates with its cognate ATPase in a symmetrical manner. Here we show that, in contrast to this general architecture, the Clp protease from *Mycobacterium smegmatis* (*Msm*) forms an asymmetric hetero-oligomeric complex ClpP1P2, which only associates with its cognate ATPase through the ClpP2 ring. Our structural and functional characterisation of this complex demonstrates that asymmetric docking of the ATPase component is controlled by both the composition of the ClpP1 hydrophobic pocket (Hp) and the presence of a unique C-terminal extension in ClpP1 that guards this Hp. Our structural analysis of ^*Msm*^ClpP1 also revealed openings in the side-walls of the inactive tetradecamer, which may represent sites for product egress.

## Introduction

Bacterial Clp proteases are generally formed by two components, a single peptidase component (ClpP) which associates with one or more members of the AAA+ (ATPases associated with a variety of cellular activities) superfamily (e.g. ClpA, ClpX or ClpC)^1–6^. In *Escherichia coli*, ClpP is expressed as a proenzyme and the N-terminal propeptide is autocatalytically removed^7^. The active complex is a barrel-shaped oligomer composed of two heptameric rings stacked back-to-back^8^. The catalytic residues (Ser-His-Asp), of this complex, are encapsulated within the barrel-shaped proteolytic chamber and access to the chamber is restricted to a narrow entry portal at either end of the complex. This design hinders entry of correctly folded proteins into the catalytic chamber, and as such prevents the indiscriminate turnover of cytosolic proteins. Substrate recognition and unfolding is mediated by the ATPase component of these machines, and ATPase docking to ClpP also couples substrate delivery to peptidase activation by triggering “gate-opening” of the peptidase entry portal^9^. ATPase docking to ClpP is mediated by two types of contacts – *static* and *dynamic*^10,11^. The primary contact is mediated by a *static* interaction between a highly conserved loop (commonly referred to as the IGF/L loop) located on the proximal face of the ATPase component, which docks into a hydrophobic pocket (Hp) on ClpP. The Hp is located at the periphery of the interface and is composed of several highly conserved aromatic and hydrophobic residues which are critical for interaction with the ATPase component^12^. The second *dynamic* contact, which involves the N-terminal loops of ClpP and the pore-2 loops of the ATPase, is axial in nature and regulated by the nucleotide state of the ATPase component^13^. The Hp is also the site of binding of a novel class of antibiotic that dysregulates ClpP function. These compounds (e.g. acyldepsipeptides (ADEPs)) not only inhibit regulated protein turnover by ClpP by blocking ATPase docking to ClpP, but they also facilitate unregulated access of cytosolic proteins into the catalytic chamber of ClpP thereby triggering their uncontrolled turnover^14–17^.

In contrast to *E. coli* several bacterial species contain two or more ClpP homologs, which form a diverse array of oligomeric complexes^18–24^. Interestingly, in *Mycobacterium tuberculosis* (*Mtb*), despite both proteins (i.e. ClpP1 and ClpP2), containing catalytic residues and a propeptide, neither protein alone is processed nor are they proteolytically active^21^. In a landmark study, Goldberg and colleagues identified Benzyloxycarbonyl-Leu-Leucinal (here termed z-LL) as a potent activator of the *Mtb* hetero-oligomeric ClpP1P2 complex, which led the group to propose that z-LL facilitated assembly of an “active” hetero-oligomer^21^. Structural analysis later confirmed that the active *Mtb* Clp protease complex was asymmetric in nature, composed of a single heptameric ring of each protein and that the activator docked into the substrate binding pocket as a substrate agonist^24^. Finally, the asymmetric nature of the ClpP1P2 complex from *Mtb* was also shown to extend to its peptidase specificity and its interaction with its cognate ATPase components^25,26^.

In this study we have examined the activation and assembly of the ClpP1P2 complex from *Mycobacterium smegmatis* (*Msm*). Similar to the ClpP1P2 complex from *Mtb*, we show that ^*Msm*^ClpP1P2 forms an asymmetric complex. The asymmetric nature of this complex is not limited to the composition of the tetradecamer but also extends to the peptidase activity of the complex (including propeptide processing), and interaction with its cognate ATPase components. In this case, our biochemical and structural analysis revealed that asymmetric docking of the ATPase components (to ^*Msm*^ClpP1P2) is controlled by two elements within ^*Msm*^ClpP1. Firstly, by a C-terminal extension in ^*Msm*^ClpP1 that obstructs access of the ATPase component to the Hp and secondly by residues that line the Hp. Together with our analysis of the ^*Ec*^ClpP Hp, we have identified Y88 (I104 in ^*Ec*^ClpP) as a key feature of ATPase docking specificity. In addition, our structure of ^*Msm*^ClpP1 reveals small openings in the side wall of the ClpP1 tetradecamer, which may represent exit portals for the egress of cleaved polypeptides.

## Results and Discussion

### Processing of the *Msm*ClpP2 propeptide by the active site residues of ^*Msm*^ClpP1 does not require an activator

**Figure 1:**
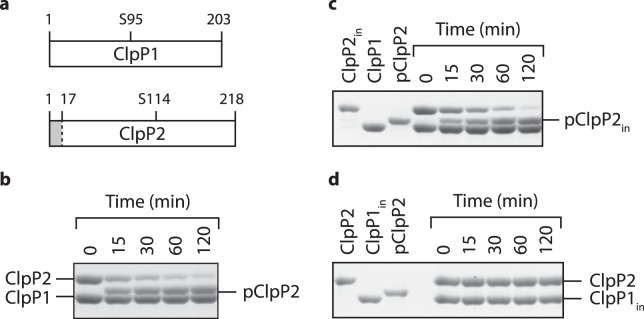
Propeptide processing of ^*Msm*^ClpP2 occurs via the active site residues of ^*Msm*^ClpP1 in the absence of the activator (z-LL). (**a**) Cartoon representation of ^*Msm*^ClpP1 and ^*Msm*^ClpP2 indicating the position of the propeptide (grey) and the catalytic Ser residues (residue 95 for ^*Msm*^ClpP1 and 114 for ^*Msm*^ClpP2). The *in vitro* processing of (**b**) wild type ^*Msm*^ClpP2 into processed (p) ^*Msm*^ClpP2 (pClpP2) or (**c**) inactive ^*Msm*^ClpP2 (ClpP2_in_) into processed ClpP2_in_ (pClpP2_in_), was monitored in the absence of activator (z-LL). (**d**) Processing of ^*Msm*^ClpP2 (in the absence of z-LL), failed to occur in the presence of the proteolytically inactive ClpP1 mutant (ClpP1_in_).

Given that correct processing of ClpP propeptides is crucial for ClpP peptidase activity^27^, we examined the processing of both ^*Msm*^ClpP components. Initially, in order to determine if both ^*Msm*^ClpP subunits were processed, we co-expressed untagged ClpP1 together with His_10_ tagged ClpP2 (ClpP2_H10_) in *E. coli* and co-purified the active ^*Msm*^ClpP1P2 complex. Although recovery of the active hetero-oligomeric complex was poor, only one component (^*Msm*^ClpP2) was processed, and this processing only occurred when the other component (ClpP1) was present (Fig. S1). To determine the location of the processing site we cloned *Msm clpP1* and *clpP2*, expressed them in *E. coli* and purified the individual components to analyse propeptide cleavage *in vitro*. Consistent with the co-expression experiment, the processing of ^*Msm*^ClpP2, required ^*Msm*^ClpP1 (Fig. 1). Interestingly, although these data are similar to the processing of the ^*Mtb*^ClpP1P2 complex, in which both ClpP subunits are processed by a hetero-oligomeric complex^21,28^ processing of ^*Msm*^ClpP2 occurred in the absence of any additional components (Fig. 1b). In contrast to the processing of ^*Msm*^ClpP2, processing of both ^*Mtb*^ClpP subunits required either the artificial activator z-LL^21^ (also see Fig. S2) or a cognate ATPase component^25^. To determine the site of processing, we performed Edman degradation of ClpP1 and processed ClpP2 (pClpP2) from the *in vitro* processing assay. Similar to ^*Ec*^ClpP and ^*Mtb*^ClpP2 (processed between Ala_12_ and Arg_13_), ^*Msm*^ClpP2 was processed between residues Ala_16_ and Arg_17_. However, in contrast to ^*Mtb*^ClpP1, which was processed between Met_7_ and Arg_8_^21,28^, no processing of ^*Msm*^ClpP1 was observed.

Next we asked, how does processing of ^*Msm*^ClpP2 occur? Initially, to address this question, we generated active site mutants of ^*Msm*^ClpP1 (ClpP1_in_) and ^*Msm*^ClpP2 (ClpP2_in_), in which the active site Ser (Ser95 and Ser114, respectively) were replaced with Ala. Similar to the processing of *Mtb* ClpP2^28^, mutation of the active site Ser in ^*Msm*^ClpP2 (^*Msm*^ClpP2_in_) did not affect processing (Fig. 1c), while in contrast processing was completely abolished by mutation of the active site Ser in ^*Msm*^ClpP1 (Fig. 1d). Collectively, these data demonstrate that processing of ^*Msm*^ClpP2 is not autocatalytic, rather it appears to occur *in trans* via the catalytic triad of ^*Msm*^ClpP1. An alternative interpretation of these data is that the catalytic triad of ^*Msm*^ClpP2 was not active. Therefore, to ensure that the catalytic triad of wild type ^*Msm*^ClpP2 was active, we examined the turnover of different model substrates (from short peptides to a folded protein), by various mixed (wild type and mutant) ClpP1P2 complexes (Fig. S3). These data demonstrated that although the catalytic triad of ClpP2 was active it was not essential for the turnover of all substrates. For example, although the active site of ClpP2 was dispensable for the turnover of short peptide substrates (Fig. S3a,b, compare lanes 2 and 6) it was necessary for efficient ^*Ec*^ClpX-mediated turnover of the model protein substrate, GFP-^*Ec*^SsrA (Fig. S3c, blue triangles). Interestingly, although mutation of ClpP1 completely inhibited the turnover of peptide substrates it had little effect on the rate of native protein turnover (Fig. S3c, red squares). In contrast mutation of ClpP2 slowed the turnover of GFP-^*Ec*^SsrA (Fig. S3c, blue triangles). Collectively, these data suggest that both ClpP1 and ClpP2 exhibit unique substrate specificities and that entry of a protein substrate (via ^*Ec*^ClpX) into the proteolytic chamber may be unidirectional.

**Figure 2:**
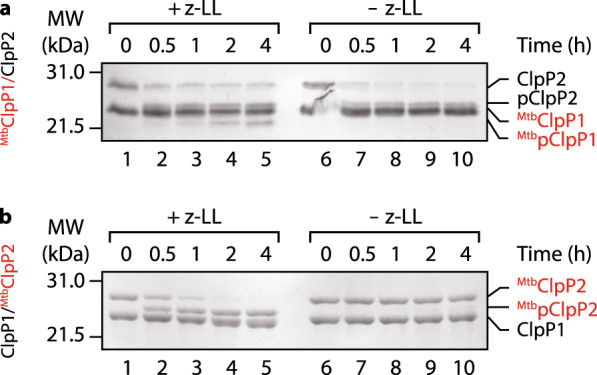
Propeptide processing of mixed ^*Mtb/Msm*^ClpP1P2 complexes in the presence or absence of z-LL. (**a**) *In vitro* processing of ^*Mtb*^ClpP1^*Msm*^P2 in the presence (lanes 1–5) or absence of z-LL (lane 6–10). (**b**) *In vitro* processing of ^*Msm*^ClpP1^*Mtb*^P2 in the presence (lanes 1–5) or absence of z-LL (lane 6–10). Following processing proteins were separated by SDS-PAGE and visualised by staining with CBB.

Next, in order to gain a better understanding of how *in trans* processing might occur we examined propeptide processing of various heterologous component combinations (Fig. 2). Initially we monitored the ability of ^*Mtb*^ClpP1 to facilitate the processing of ^*Msm*^ClpP2 in the absence or presence of z-LL (Fig. 2a). Interestingly, consistent with the processing of ^*Msm*^ClpP2 by ^*Msm*^ClpP1 (Fig. 1b), z-LL was not required for the ^*Mtb*^ClpP1-mediated processing of ^*Msm*^ClpP2, despite an absolute requirement of z-LL for all ^*Mtb*^ClpP1 activities (Fig. S2). Furthermore, the processing of ^*Mtb*^ClpP1 was not required for processing of ^*Msm*^ClpP2 to occur. Hence these data suggest that the initial processing event (of ClpP2) may occur by a mechanism that is distinct from the downstream processing of ClpP1. We then monitored the ability of ^*Msm*^ClpP1 to facilitate the processing of ^*Mtb*^ClpP2 (Fig. 2b). Surprisingly, despite the fact that z-LL was not required for the ^*Msm*^ClpP1-mediated processing of ^*Msm*^ClpP2, it (z-LL) was essential for the processing of ^*Mtb*^ClpP2. This suggests that z-LL is required to trigger a conformational change in ^*Mtb*^ClpP2 that mediates its processing by ^*Msm*^ClpP1.

### ^*Msm*^ClpP2 is processed via a transient complex

**Figure 3:**
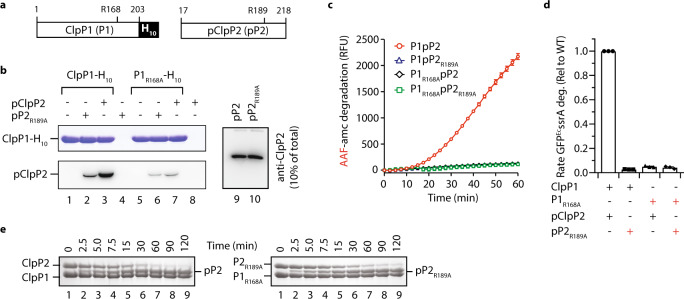
In contrast to peptide and protein degradation, propeptide processing of ^*Msm*^ClpP2 does not require stable interaction between ^*Msm*^ClpP1 and ^*Msm*^ClpP2. (**a**) Cartoon representation of ^*Msm*^ClpP1-H_10_ and ^*Msm*^ClpP2 indicating the position of the conserved Arg finger in ClpP1 (R168) and ClpP2 (R189). (**b**) Co-immunoprecipitation (co-IP) of wild type (lanes 1–3) or mutant ClpP1-H_10_ (lanes 5–7) in the presence of either wild type (lanes 3 and 7) or mutant pClpP2 (lane. 2 and 6). The total amount of wild type or mutant pClpP2 (lane 9 and 10, respectively) added to the co-IP is indicated. (**c**–**d**) The turnover of AAF-amc peptide (c) or rate of GFP-^*Ec*^ssrA degradation was monitored by fluorescence using wild type or mutant ClpP1P2 complexes (**e**) comparison of propeptide processing by the wild type ClpP1P2 complex (left panel) and the ClpP1_R168A_P2_R189A_ complex (right panel).

Although propeptide processing of homo-oligomeric ClpP complexes such as ^*Ec*^ClpP and *Homo sapiens* ClpP (^*Hs*^ClpP) has long been described as autocatalytic^7^, the mechanism by which this step occurs is poorly defined and currently it remains unclear if processing occurs before (or as a result of) assembly of the tetradecamer, or indeed if processing occurs via the *cis* or *trans* ring^7,27,29^. Therefore, we examined the composition of the active “processing” complex. For homo-oligomeric ClpP complexes, it is plausible that processing is mediated by the *cis* ring and the processing site is determined via a molecular “ruler” mechanism. For hetero-oligomeric complexes such as ClpP1P2^24^, the distance between the processing site (within the propeptide) and the active site within the *trans* ring (~47 Å) is estimated to be much greater than the distance between the propeptide and the active site in the *cis* ring (~25 Å). Hence, to determine if processing of ClpP2 is mediated by formation of the ClpP1P2 complex, we generated single point mutations in both ^*Msm*^ClpP proteins to disrupt the ring-ring interface. This mutation was based on a critical Arg residue that stabilises the ring-ring interface of *Staphylococcus aureus* ClpP (^*Sa*^ClpP)^30^. Specifically, we replaced the Arg-finger residue in ^*Msm*^ClpP1 and ^*Msm*^ClpP2 (Arg168 and Arg189, respectively) with Ala. Initially, to ensure the overall structure of each mutant protein was not compromised, we compared the oligomeric state of each protein in the absence of z-LL using analytical ultracentrifugation (AUC). Importantly, both wild type and mutant ^*Msm*^ClpP1 and ^*Msm*^ClpP2 each formed heptamers (Fig. S4, red circles). Next, to monitor the effect of these mutations on ^*Msm*^ClpP1P2 complex formation we performed a series of pull-down experiments, in which wild type or mutant ^*Msm*^ClpP1_H10_ was immobilised to Ni-NTA-agarose beads. Importantly, mutation of a single component was sufficient to significantly reduce its interaction with the other component (Fig. 3), while mutation of both components almost completely abolished the interaction of the two components (Fig. 3b, lane 6). These data demonstrate that, similar to ^*Sa*^ClpP, the Arg finger plays a crucial role in stabilising the ^*Msm*^ClpP1P2 complex. Consistent with this loss of the ClpP1P2 tetradecamer, the turnover of a model peptide (Fig. 3c) or protein (Fig. 3d) substrate, by each of the different mutant protein complexes, was completely abolished. Surprisingly, and in contrast to the peptidase activity of each mutant protein complex, all three complexes retained the ability to process ClpP2, with only a modest change to the rate of processing (Fig. 3e). One explanation for these data is that the substrate (i.e. the propeptide) is, in this case, tethered to the peptidase and hence any residual interaction between the two rings may be sufficient to facilitate rapid cleavage of the propeptide. An alternative explanation for these data is that processing of ClpP2 is not mediated by the classic ClpP1P2 tetradecamer, but rather by an interaction that does not require inter-ring contacts mediated by the Arg fingers. Consistent with this idea, propeptide processing of ^*Msm*^ClpP2 (by ^*Msm*^ClpP1) is independent of z-LL activity (Fig. 1), while in contrast the activator is essential for all other proteolytic activities of the ^*Msm*^ClpP1P2 complex.

### Mutation of the hydrophobic pocket modulates the peptidase activity of the ^*Msm*^ClpP1pP2 complexes

Next, in order to study the interaction of the ^*Msm*^ClpP1P2 complex with its cognate ATPase components (ClpX and ClpC1), we generated specific point mutations within the hydrophobic pockets of ^*Msm*^ClpP1 and ^*Msm*^ClpP2. Initially, we targeted two residues in the Hp (the first was a highly conserved tyrosine residue found in all ClpP sequences, Y60 in ClpP1 and Y79 in ClpP2, the second hydrophobic residue was less conserved across ClpP sequences, Y110 in ClpP1 and L129 in ClpP2). Each of the hydrophobic residues (described above) was replaced with alanine, to generate a series of single point mutants (P1_Y60A_, P1_Y110A_, P2_Y79A_ or P2_L129A_) and one double mutant of ^*Msm*^ClpP2 in which both hydrophobic residues (Y79 and L129) were replaced with alanine, here referred to as P2_dbl_. To ensure that mutation of the Hp of ^*Msm*^ClpP1 and ^*Msm*^ClpP2, did not alter the overall structure of each protein, we examined the oligomeric state of each Hp mutant using AUC and compared them to the wild type proteins (Fig. S4). Importantly, with the exception of P1_Y60A_, the oligomeric state (as determined by AUC) of each mutant was largely unaffected (Fig. S4). A similar trend for ClpP1 mutants was also observed using Native-PAGE (Fig. S4), although in this case, again with the exception of P1_Y60A_ the gel appeared to stabilize the 14-mer. Stabilisation of the 14-mer by Native-PAGE has also been observed for human ClpP^29^. Surprisingly, neither the 7-mer nor the 14-mer complexes of ClpP2 were observed in Native-PAGE.

Next, we compared the peptidase activity of the wild type ^*Msm*^ClpP1P2 complex with the various ^*Msm*^ClpP1 and ^*Msm*^ClpP2 mutant proteins (Fig. S5). Interestingly, despite the changes to the oligomeric state of P1_Y60A_ only a small change in the rate of AAF-amc turnover (by P1_Y60A_P2) was observed (Fig. S5a), while in contrast, the rate of LY-amc turnover by P1_Y60A_P2 was unexpectedly increased by ~4-fold (Fig. S5b). Collectively these data appear to suggest that the replacement of aromatic residues (e.g. Y60A) within the Hp of ClpP1 may affect the conformation of the substrate binding pocket (S1) directly, which in the presence of the peptide agonist z-LL, could modulate substrate affinity, specificity and/or cleavage. Consistently, ClpP1 is directly responsible for the turnover of both peptide substrates, as replacement of the active site Ser of ^*Msm*^ClpP1 with Ala (P1_in_) abolished the turnover of both substrates (Fig. S3). In contrast to P1_Y60A_ the relative peptidase activity of P1_Y110A_ (by the P1_Y110A_P2 complex) was essentially unchanged for both substrates (Fig. S5a,b). Importantly, given that P1_Y110A_ did not exhibit any substrate-dependent defects in peptidase activity, we limited our subsequent analysis to the ClpP1_Y110A_P2 complex. Next, we examined the effect of the different Hp mutations in ^*Msm*^ClpP2. Surprisingly, the peptidase rate for both substrates was significantly reduced (by up to 80%) for both single point mutants (Y79A and L129A) of ^*Msm*^ClpP2 (P2_Y79A_ and P2_L129A_) (Fig. S5c,d). Intriguingly, the rate of peptide degradation by the double mutant (P2_dbl_) was unchanged for both substrates (Fig. S5c,d, compare columns 1 and 4). Currently it remains unclear why the peptidase activity of the two single mutants in complex with wild type ClpP1 is reduced. However, given that the double mutant exhibited a similar peptidase activity to the wild type complex, all further analysis was limited to the ClpP1P2_dbl_ complex.

### ^*Msm*^ClpP1P2 forms asymmetric complexes with its cognate ATPase

**Figure 4:**
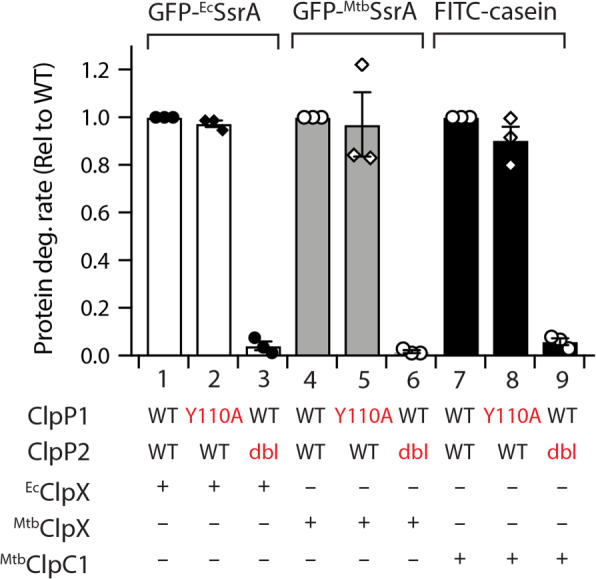
^*Msm*^ClpP1P2 form an obligate single-headed complex with its cognate ATPase components. (**a**) The ^*Ec*^ΔNClpX-mediated degradation of GFP-^*Ec*^SsrA is mediated by docking to ^*Msm*^ClpP2. Although mutation of the Hp residue (Y110) to Ala did not affect the ATPase-mediated delivery of GFP-^*Ec*^SsrA (red squares), mutation of the Hp residues (Y79 and Y129) abolished turnover (blue triangles). (**b**) The rate of either GFP-^*Ec*^SsrA degradation by ^*Ec*^ΔNClpX (white bars), GFP-^*Mtb*^SsrA degradation by ^*Mtb*^ClpX (grey bars) or FITC-casein degradation by ^*Mtb*^ClpC1 (black bars) was determined from three independent experiments (n = 3) using either wild type ClpP1P2 (lanes 1, 4 and 7), ClpP1_Y110A_P2 (lanes 2, 5 and 8) or ClpP1P2_dbl_ (lanes 3, 6 and 9). Error bars represent SEM.

To determine the mode of ATPase docking to the *Msm*ClpP1P2 complex, we examined the degradation of several ATPase-dependent substrates in the presence of wild type or mutant complexes of ^*Msm*^ClpP1P2. Initially, we examined the ATPase-dependent turnover of GFP-^*Ec*^SsrA by Hp mutants of ^*Msm*^ClpP1 (in the presence of wild type ClpP2) using the non-cognate ATPase, ^*Ec*^ΔNClpX. Consistent with recent findings^31^, ^*Ec*^ΔNClpX was able to mediate the turnover of GFP-^*Ec*^SsrA by ^*Msm*^ClpP1P2, either in the absence or presence of the peptide activator, z-LL. Significantly, despite differences in peptidase activity of the various ClpP1 Hp mutants (Fig. S5) the ^*Ec*^ΔNClpX -mediated turnover of GFP-^*Ec*^SsrA by each mutant protein complex was equivalent to the wild type complex (Fig. S6). Next, we examined the ^*Ec*^ΔNClpX-mediated turnover of the same substrate by ClpP1P2 complexes bearing Hp mutations in ClpP2 (Fig. S6). Importantly, mutation of either or both Hp residues on ClpP2 abolished the ATPase-mediated turnover of GFP-^*Ec*^SsrA (Fig. S6). Collectively, these data suggest that the ATPase component docks exclusively to ClpP2. To confirm the asymmetric nature of the ^*Msm*^ClpP1P2 complex we monitored the turnover of two additional model substrate that are mediated by Mycobacterial ATPase components (i.e. GFP-^*Mtb*^SsrA for ClpX and fluorescently-labelled model unfolded protein, FITC-casein for ClpC1). In this case, given that the “ClpP docking loop” of each ATPase component (ClpX and ClpC1) is conserved across *Msm* and *Mtb* (Fig. S7), the turnover of each substrate by the various Hp mutant complexes was examined in the presence of either ^*Mtb*^ClpX or ^*Mtb*^ClpC1, respectively. Consistent with the asymmetric binding observed for ^*Ec*^ΔNClpX, the ^*Mtb*^ClpX-dependent turnover of GFP-^*Mtb*^SsrA was unaffected by mutation of the Hp in ^*Msm*^ClpP1 (Fig. 4 lane 5). In contrast, the equivalent Hp mutation in ^*Msm*^ClpP2 completely abolished substrate turnover (Fig. 4, lane 6). Similarly, the ^*Mtb*^ClpC1-dependent turnover of FITC-casein was unaffected by mutation of the Hp in ^*Msm*^ClpP1 (Fig. 4, lane 8), while the equivalent Hp mutation in ^*Msm*^ClpP2 effectively abolished the turnover of FITC-casein (Fig. 4, lane 9). Collectively these data indicate that, mutation of the Hp in ^*Msm*^ClpP2 is sufficient to completely abolish the turnover of substrates by all ATPases tested. Indeed, both the cognate ATPases (^*Mtb*^ClpX and ^*Mtb*^ClpC1) and the heterologous ATPase (^*Ec*^ClpX) bind specifically to ^*Msm*^ClpP2 and not to ^*Msm*^ClpP1 forming single-headed, asymmetric complexes (ClpP1P2X and ClpP1P2C1). These data are consistent with the findings by Weber-Ban and colleagues who showed that the ^*Mtb*^ClpP1P2 complex only binds to its partner unfoldase components through ClpP2^25^.

**Figure 5:**
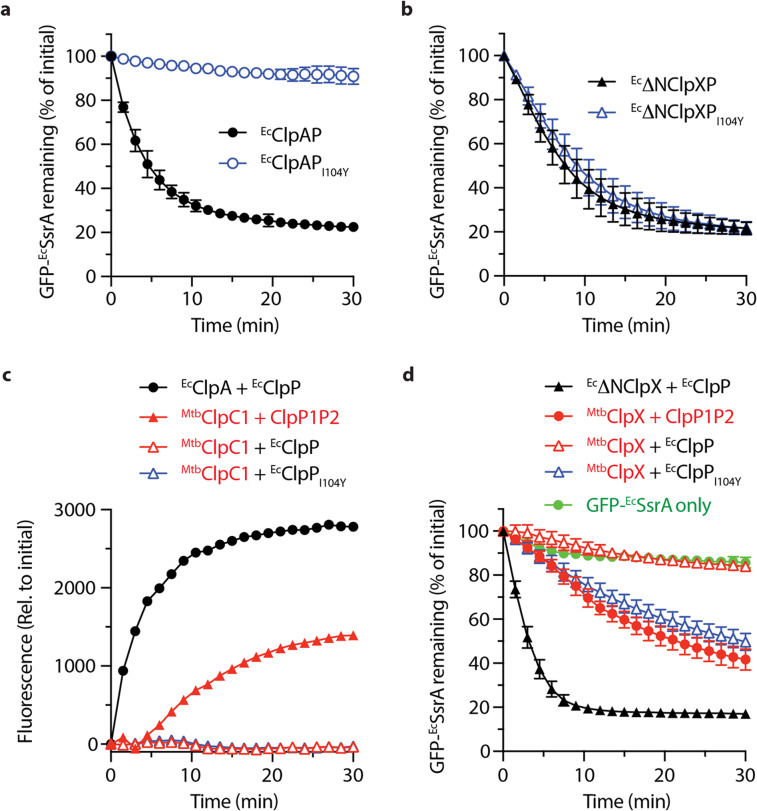
Single point mutations in the Hp of ^*Ec*^ClpP inhibit ClpA-mediated substrate-turnover but facilitate ^*Mtb*^ClpX-mediated substrate-turnover. The degradation of GFP-^*Ec*^SsrA mediated by (**a**) ^*Ec*^ClpA and (**b**) ^*Ec*^ΔNClpX was monitored by fluorescence in the presence of wild type ^*Ec*^ClpP (black symbols) or ^*Ec*^ClpP_I104Y_ (blue symbols). (**c**) The degradation of FITC-casein was monitored by fluorescence in the presence of either ^*Ec*^ClpAP (filled circles) or ^*Mtb*^ClpC1 (triangles) together with ^*Msm*^ClpP1P2 (filled triangles), ^*Ec*^ClpP (open red triangles) or ^*Ec*^ClpP_I104Y_ (open blue triangles). (**d**) The turnover of GFP-^*Ec*^SsrA was monitored by fluorescence in the absence of any addition (green circles) or in the presence of either ^*Ec*^ΔNClpXP (filled black triangles) or ^*Mtb*^ClpX together with either ^*Msm*^ClpP1P2 (filled red circles), ^*Ec*^ClpP (open red triangles) or ^*Ec*^ClpP_I104Y_ (open blue triangles). Degradation rates were determined from three independent experiments (n = 3). Error bars represent SEM.

Next, to better understand the molecular basis of this asymmetric specificity we compared the Hp residues of several ClpP homologs. From this analysis we noticed that in contrast to most ClpP sequences, ^*Msm*^ClpP1 contained an additional aromatic residue (Y88) within the Hp and speculated that this aromatic residue inhibited ATPase docking (Fig. S8). To test this hypothesis, we first replaced the equivalent residue in ^*Ec*^ClpP (I104) with tyrosine to generate ^*Ec*^ClpP_I104Y_ and tested the ability of this mutant protein to interact with its cognate ATPase components (ClpA and ClpX). Consistent with the idea that Y88 is a crucial inhibitory element within the Hp, ^*Ec*^ClpP_I104Y_ prevented the ClpA-mediated degradation of GFP-^*Ec*^SsrA (Fig. 5a). However, the same mutation had no effect on the ClpX-mediated turnover of SsrA-tagged GFP (Fig. 5b). Interestingly, although the introduction of tyrosine at residue 104 (in ^*Ec*^ClpP) was sufficient to inhibit ClpA docking, ^*Ec*^ClpP (bearing Ile at residue 104) was unable to functionally interact with either ^*Mtb*^ClpC1 (Fig. 5c, open red triangles) or ^*Mtb*^ClpX (Fig. 5d, open red triangles). In contrast to ClpA docking, replacement of Ile104 with Tyr in ^*Ec*^ClpP (^*Ec*^ClpP_I104Y_), did not affect ^*Ec*^ClpX-docking. However, this mutation was sufficient to recover wild type-like activity with ^*Mtb*^ClpX. Collectively these data indicate that tyrosine (at residue 104/88) although inhibitory to ClpA and ClpC1 docking is permissive to ClpX docking. This in turn suggests that residue 104/88 may act as a “sensor” of ATPase docking and not simply a key inhibitory element within ^*Msm*^ClpP1, that obstructs ATPase docking.

**Figure 6:**
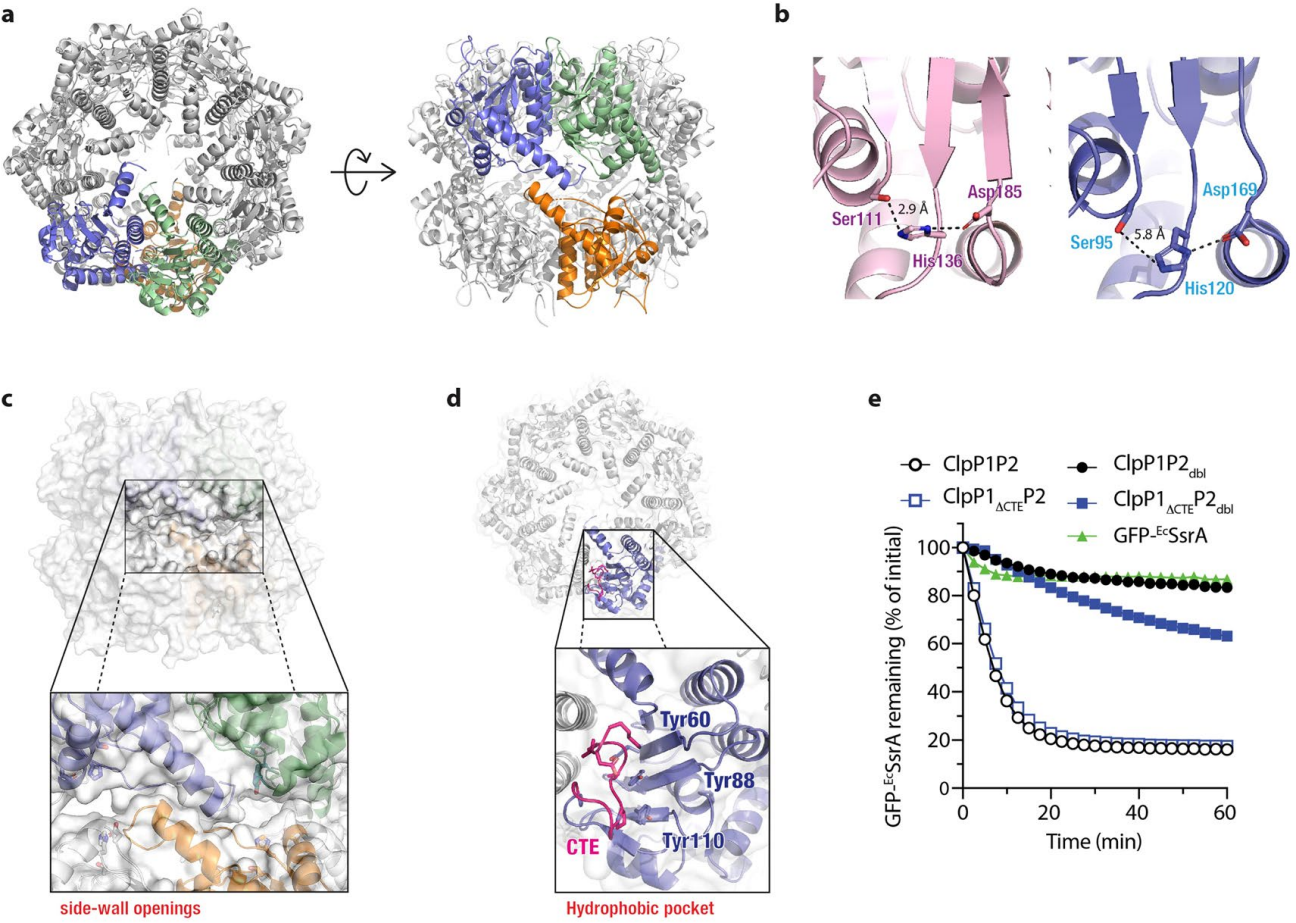
The C-terminal extension (CTE) of ^*Msm*^ClpP1 obstructs ATPase docking and substrate delivery. (**a**) Ribbon representation of the oligomeric tetradecamer of ^*Msm*^ClpP1 in top view (left panel) and side-view (right panel). Individual subunits are indicated in green, blue and orange. (**b**) Close up view of the catalytic triad of ^*Msm*^ClpP1 (slate blue) in comparison to ^*Ec*^ClpP (light pink, PDB code 3MT6) demonstrating that the His120 is dislocated ~5.8 Å from the catalytic Ser residue (Ser 95) (**c**) Surface representation of ^*Msm*^ClpP1 tetradecamer highlighting side-wall openings located between the rings, adjacent to the catalytic triad. (**d**) Three tyrosine residues (Tyr60, Tyr88 and Tyr110) line the Hp and interact with the CTE (pink). (**e**) The ^*Ec*^ΔNClpX-mediated degradation of GFP-^*Ec*^SsrA was monitored by fluorescence in the presence of various ClpP1P2 complexes. Although the turnover of GFP-^*Ec*^SsrA by wild type ClpP1P2 (open black circles) was not affected by deletion of the CTE (open blue squares) it was blocked by mutation of the Hp in ClpP2 (filled black circles). Importantly, degradation via the ClpP1P2_dbl_ was recovered when the CTE was removed from ClpP1 (filled blue squares). Degradation assays were determined from three independent experiments (n = 3). Error bars represent SEM.

### Structural basis for docking asymmetry of *Msm*ClpP1P2

To better understand the molecular features that define ATPase docking we crystallised ^*Msm*^ClpP1 (Fig. 6). The structure of ^*Msm*^ClpP1 was determined to 2.0 Å resolution and refined to an *R*_free_ value of 21.5% (Table 1). Similar to other ClpP structures, ^*Msm*^ClpP1 was composed of two heptameric rings stacked back-to-back (the asymmetric unit consisted of one heptamer, the biological tetradecamer is formed from crystallographically related heptamers). The tetradecamer forms a compact barrel-shaped oligomer with approximate dimensions 86 × 102 Å (Fig. 6a). The overall fold of the ^*Msm*^ClpP1 protomer is similar to most ClpP structures; structural superposition of ^*Msm*^ClpP1 with ^*Mtb*^ClpP1 (2CBY)^32^, ^*Mtb*^ClpP1P2 (4U0G)^24^ and ^*Ec*^ClpP (3MT6)^17^ resulted in overall r.m.s.d. values of 0.8 Å (1183 Cα aligned), 2.3 Å (1024 Cα aligned) and 2.6 Å (1071 Cα aligned), respectively. However, there are a number of notable differences between these structures. Firstly, the structure of ^*Msm*^ClpP1, similar to ^*Mtb*^ClpP1^32^, is in an inactive conformation, as His120 is dislocated from the catalytic triad (i.e. the distance between Ser95 and His120 is 5.8 Å (Fig. 6b, right panel) (6.55 Å for the equivalent residues ^*Mtb*^ClpP1). In contrast, in the ClpP1 component of the ^*Mtb*^ClpP1P2 complex (4U0G) and in ^*Ec*^ClpP, these residues are only 2.9 Å apart (Fig. 6b, left panel). Another key difference between these structures relates to the formation of the tetradecamer, via the handle domains. In ^*Ec*^ClpP and ^*Mtb*^ClpP1P2 complex, the two heptameric rings associate via the formation of an antiparallel β-sheet in the handle domains, whereby each β-sheet is composed of strands from opposing monomers across the ring-ring interface. Compared to these active structures, both ^*Msm*^ClpP1 and ^*Mtb*^ClpP1, have shorter handle domains, which lack a structured β-strand region. As a result, association of the heptameric rings in ^*Msm*^ClpP1 (and ^*Mtb*^ClpP1) is mediated largely by a *trans* association of a short section of the α-helix in the handle domain creating several openings in the side-walls of the ClpP1 tetradecamer (Figs. 6c and S9). Although handle flexibility has been observed in a selection of inactive ClpP structures, the size and precise location of pores resulting from this flexibility has been difficult to assess due to the large number of unmodeled residues in these structures. Significantly, the breaches in the side wall of ^*Msm*^ClpP1 are located adjacent to the catalytic residues of the protease and as such provide a direct path between the active site of the protease and the external solution. Hence as has been proposed by Kay and Houry^33^, these openings could represent an exit portal for the egress of peptides. Therefore, we postulate that this structure of ^*Msm*^ClpP1 represents a post “substrate-cleavage” snapshot of the protease. Following peptide cleavage, this protease acquires an inactive conformation where the catalytic His residue adopts a distorted configuration, which initiates a conformational change in the adjacent handle domain whereby the β-strand that typically forms an antiparallel β-sheet with the equivalent β-strand in the opposite monomer, becomes disordered. As a result of this disorder in the β-strand, several interactions which stabilise the tetradecamer are lost. This results in a shorter interface between opposing subunits in the tetradecamer and opening of “large pores” along the surface of the protein which could allow release of the cleaved peptides. Smaller solvent exposed channels have also been observed in the side walls of the ^*Mtb*^ClpP1P2 complex (Fig. S9), however these openings were only located between ClpP1 subunits, above the equatorial interface, ~15 Å from the catalytic triad^24^.

**Table 1:** Crystallographic data and refinement statistics.

Data collection	
**PDB ID: 6BPU**	
Wavelength (Å)	0.9537
Oscillation, total degrees (°)	(1°/180°)
Resolution range (Å)^*^	50.0–2.00 (2.03–2.00)
Space group	*P*4_2_2_1_2
Unit-cell parameters (Å, °)	α = β = 169.62; γ = 114.22 α = β = γ = 90
No. of molecules in asymmetric unit	7
Observed reflections	1386, 431
Unique reflections	111, 562
Rpim	0.035 (0.364)
Completeness (%)^*^	99.7 (98.3)
‹*I/σ(I)*›^*^	9.29 (2.9)
Multiplicity^*^	12.4 (11.0)
**Refinement**	
Resolution (Å)^*^	37.15–2.00 (2.07–2.00)
R_factor_^a*^	0.178 (0.224)
R_free_^b*^	0.214 (0.260)
No. of non-hydrogen atoms	
- Protein	10172
- Waters	1087
- Malonate (molecules)	63
B factors	
- Wilson (Å^2^)	26.68
- Average B factor (Å^2^): All atoms	31.32
- Average B factor (Å^2^): Macromolecules	30.29
- Average B factor (Å^2^): Ligands	51.60
- Average B factor (Å^2^): Solvent	39.80
R.m.s.d. from ideal geometry	
- Bonds (Å)	0.007
- Angles (°)	0.88
MolProbity analysis	
- Ramachandran favored/outliers (%)	96.50/0.3
- Clashscore [percentile]	5.21 (97th)

^*^Values in parentheses refer to the highest resolution shell.

^a^*R*fac = Σh |Fo − Fc|/Σh|Fo|, where Fo and Fc are the observed and calculated structure-factor amplitudes for each reflection “h”.

^b^*R*free was calculated with 5% of the diffraction data selected randomly and excluded from refinement.

Another distinctive feature of ^*Msm*^ClpP1, which is absent in many ClpP homologues including ^*Mtb*^ClpP1^24,32^, is a short C-terminal extension (CTE) ~12 residues long, which docks into its own Hp (Fig. 6d). Interestingly, the CTE interacts with residues within the Hp of ClpP in a similar manner to ADEP^16,17,24^. Specifically, the CTE interacts with three tyrosine residues (Y60, Y88 and Y110) within the Hp (Fig. 6d). Hence, we speculated that, similar to ADEPs, the CTE of ^*Msm*^ClpP1 may restrict ATPase docking. To test this idea, we deleted the CTE of ^*Msm*^ClpP1 and monitored the ability of various wild type and mutant ^*Msm*^ClpP1P2 complexes to interact with different ATPase components (Figs. 6e and S10). First, we examined the ^*Ec*^ClpX-mediated turnover of GFP-^*Ec*^SsrA by ^*Msm*^ClpP1_ΔCTE_ in the presence of wild type ^*Msm*^ClpP2. Importantly, this complex retained wild type-like activity against each ATPase/substrate combination tested, demonstrating that removal of the CTE does not alter ClpP1P2 activity (Fig. 6e, open symbols). Next, in order to switch ATPase-docking specificity (from ^*Msm*^ClpP2 to ^*Msm*^ClpP1), we repeated the above experiments in the presence of ^*Msm*^ClpP2_dbl_ (which is unable to dock to any of the ATPase components tested). Remarkably, deletion of the CTE was sufficient to facilitate the ^*Ec*^ClpX-mediated turnover of GFP-^*Ec*^SsrA (Fig. 6e, filled squares). These data demonstrate that the CTE of ^*Msm*^ClpP1 plays a crucial inhibitory role in ATPase docking. Next, we monitored the docking of the cognate ATPase components (^*Mtb*^ClpX and ^*Mtb*^ClpC1) to the ClpP1_ΔCTE_P2_dbl_ complex. Surprisingly, neither ^*Mtb*^ClpX nor ^*Mtb*^ClpC1 were able to mediate the turnover of either GFP-^*Mtb*^SsrA (Fig. S10b) or FITC-casein (Fig. S10c) respectively. Taken together, these data suggest that although the CTE obstructs ATPase docking (of ^*Ec*^ClpX), removal of this feature alone is not sufficient to permit docking to the physiological relevant ATPase components (^*Mtb*^ClpX/^*Mtb*^ClpC1). Hence, both the presence of the CTE (that occludes the Hp of ClpP1) and the altered specificity of the Hp may have important implications for the development of novel ADEP-like antibiotics that dysregulate Clp proteases.

Interestingly, several ClpP homologs contain a C-terminal extension (including human ClpP which contains a 28-residue extension). To date however, the role of these CTEs has remained unclear. In contrast to our structure of ^*Msm*^ClpP1, the CTE of ^*Hs*^ClpP extends away from the heptameric ring^27^. Despite this “snapshot” placing the CTE of human ClpP away from the ATPase-interface we speculate that this region (in various ClpP homologues) could play an important role in regulating ClpP function *in vivo*. One possibility is that the CTE (or in the case of ^*Msm*^ClpP1 - the atypical Hp) may have co-evolved with an alternate Clp-protease activator to further diversify the function of the peptidase. Consistent with this idea, novel (ATP-independent) Clp protease activators have recently been identified in plants^34^. Likewise, the Mycobacterial proteasome has also been shown to function with a variety of activators (both ATP-dependent and ATP-independent)^3,35–38^. Therefore, we speculate that novel ClpP activators may exist in a variety of species which contain ClpP homologs with either an extended C-terminus or an atypical Hp.

In conclusion, our findings clearly demonstrate that, similar to ^*Mtb*^ClpP1P2, the hetero-oligomeric Clp-protease complex in *Msm* is highly regulated. Although processing of ^*Msm*^ClpP2 (and hence “activation” of the ^*Msm*^ClpP1P2 complex) can proceed in the absence of an *activator*, the *in vitro* peptidase activity of ^*Msm*^ClpP1P2 (for peptide and protein turnover) requires either a cognate ATPase component or a chemical activator (i.e. z-LL). Significantly, both cognate ATPase components dock to only one face of the peptidase. This asymmetry provides direct competition for ATPase-docking to the peptidase, and as a result (dependent on the abundance of each component), likely controls the delivery of specific substrates to this peptidase and hence their turnover in a tightly regulated fashion. The specific docking of both ATPase components to a single face of this machine may also provide an opportunity for further diversification of the peptidase, through docking of additional specific activators to the vacant platform on the ClpP1P2 complex.

## Material and Methods

### Cloning

*Msm clpP1* (MSMEG_4673) and *clpP2* (MSMEG_4672) were amplified from *M. smegmatis* mc^2^155 genomic DNA (kindly provided by Prof. R. Manganelli) with specific primers (see Table S1), using Phusion DNA polymerase (New England Biolabs). *Mtb clpP1* (Rv2460c) and *clpP2* (Rv2461c) were amplified from *Mtb* genomic DNA (kindly provided by Ms. M. Globan, VIDRL). The amplified DNA was digested with the appropriate restriction enzymes and ligated into similarly digested plasmids. Fragments coding for unprocessed ClpP1 and ClpP2 were cloned into pET10C (and pET10N)^39^ to generate either a C- or N-terminal His_10_-tagged fusion protein, while fragments coding for processed forms of ClpP1 and ClpP2 (i.e. lacking propeptide) were cloned into pHUE^40^ to generate N-terminal His_6_-Ub fusion proteins. *Mtb clpX* cloned into pET15(b) was a kind gift from Dr. P. Genevaux (Université de Toulouse, France), and *Mtb* *clpC1* cloned into pET30(a) was a kind gift from Dr. D. Vasudevan (Institute of Life Sciences, Bhubaneswar, India)^41^. The SsrA-tag from *Mtb/Msm* (AADSNQRDYALAA) was cloned into pDD173^42^ by annealing specific primers (see Table S1) to generate a C-terminal GFP fusion (GFP-^*Mtb*^SsrA). All clones (Table S2) were verified by nucleotide sequencing.

### Protein expression and purification

His_6_-tagged ^*Ec*^ClpX and ^*Ec*^ClpP were expressed in *E. coli* and purified as described previously^43^. ^*Mtb*^ClpC1 and ^*Mtb*^ClpX were both expressed as C-terminal His_6_-tagged fusion proteins. ^*Mtb*^ClpC1-His_6_ was expressed as described in^41^, while ^*Mtb*^ClpX-His_6_ was expressed at 16 °C in *E. coli* BL21 (DE3) codon+ RIL cells, following the addition of 0.5 mM IPTG. ClpP1 and ClpP2 (from either *Msm* or *Mtb*) were expressed, with either a C-terminal His_10_ tag, essentially as described in^39^ or alternatively with a His_6_-Ub tag (which was subsequently cleaved) as described^44^. All His_6_-tagged fusion proteins were purified using Ni-NTA-agarose beads as described previously^43^, while His_10_-tagged fusion proteins were purified essentially as described^45^. Untagged ClpP1 and ClpP2 (wild type and specific point mutants) were also generated using the Ub-fusion system^40^ and purified essentially as described, using a combination of IMAC and preparative grade size exclusion chromatography (SEC).

### Protein analysis by electrophoresis

For the analysis of protein purity and protein turnover, samples were separated by 16.5% Tricine SDS-PAGE^46^. To analyse the oligomerisation/native structure of wild type and mutant ClpP1 complexes, 5 µg of purified protein was separated using 4–16% Native-PAGE Novex Bis-Tris gels (Invitrogen) essentially as described^29^ and visualised by staining with Coomassie Brilliant Blue (CBB) R250.

### Processing and peptidase assays

For processing assays, either full length *Msm* or *Mtb* ClpP1 was incubated together with full length ClpP2 (from either *Msm* or *Mtb*) at 30 °C in Buffer XP (25 mM Tris-HCl pH 7.5, 100 mM KCl, 100 mM NaCl, 20 mM MgCl_2_, 10% (v/v) glycerol, 0.025% (v/v) Triton X-100, 1 mM DTT) in the absence or presence of the activator, z-Leu-Leu-H (z-LL). Processing of ClpP (2.8 µM) was initiated, either by addition of the activator (0.5 mM), or equimolar amounts of the partner ClpP protein. The reaction was stopped (at the indicated time points), by the addition of sample buffer followed by incubation at 95 °C for 5 min. Processing of ^*Msm*^ClpP was analysed by 16.5% Tris-tricine SDS-PAGE while processing of ^*Mtb*^ClpP was analysed by 15% glycine SDS-PAGE. To monitor the peptidase activity of wild type and mutant ClpPs, peptide degradation assays were performed using fluorescently labelled peptides essentially as described^29^. Similarly, the turnover of GFP-^*Ec*^SsrA, GFP-^*Mtb*^SsrA or FITC-casein (by 1 µM ^*Ec*^ΔNClpX, ^*Mtb*^ClpX or ^*Mtb*^ClpC1, respectively) was and monitored by fluorescence as described^29^. All peptide and protein degradation assays were performed in the presence of 0.5 mM z-LL (unless otherwise stated).

### Crystallisation of ^*Msm*^ClpP1 and diffraction data collection

Purified ^*Msm*^ClpP1 (in 50 mM Tris-HCl pH 8.0, 200 mM KCl) was concentrated using a Centricon-30 centrifugal concentrator (Amicon) up to 30 mg/mL. ^*Msm*^ClpP1 crystallization experiments were performed using the vapor diffusion method. Initial high-throughput crystallization experiments were performed in house or at the CSIRO Collaborative Crystallization Centre (www.csiro.au/C3; Melbourne, Australia). For crystal optimization experiments, drops were set in 24-well plates by mixing 1 µL of protein with 1 µL of well condition and drops were equilibrated at 20 °C against a reservoir volume of 500 µl. Small hexagonal crystals were obtained in 1.8M sodium malonate pH 6.4. After two rounds of optimisation, which included changing pH, malonate concentration and protein concentration a significant improvement in the quality of the crystals was obtained. Larger hexagonal crystals (with approximate dimensions 0.3 mm × 0.2 mm × 0.1 mm) were obtained from solutions consisting of 2.1–3.4M sodium malonate pH 6.6–6.8 and a protein concentration of 5.5 mg/mL.

The crystals were cryoprotected using 3M sodium malonate pH 6.5–6.9 and flash cooled in liquid nitrogen. Diffraction data was collected at 100 K at the protein crystallography beamline MX2 at the Australian synchrotron using an ADSC Quantum 315r detector. 1° oscillation images were collected for a total of 180° using a crystal-to-detector distance of 260 mm. Diffraction data were integrated and scaled with HKL2000^47^.

### Structure determination and refinement

The crystal structure of ^*Msm*^ClpP1 was solved by molecular replacement using BALBES^48^ using the structure of *M. tuberculosis* ClpP as a search model (PDB: 2CBY, sequence identity 96%). The model was built using Coot^49^ and refined using phenix.refine^50^ and TLS (translation/libration/screw) refinement^51^. Most of the structure could be unambiguously assigned in the electron density map except residues 1–10 at the N- terminus of each chain, and the loop region between residues 125–129, which in chains A, B, C and E were difficult to model because of poor density. The final model was validated using Molprobity^52^. Table 1 provides the statistics for the X-ray data collection and final refined model. All structural figures were generated with PyMOL. Superposition of molecules was carried out using the Secondary Structure Matching (SSM) option from the program Coot^49^.

## Supplementary information

Supplementary Information
