# Supplementary material for: Molecular and structural insights into an asymmetric proteolytic complex (ClpP1P2) from Mycobacterium smegmatis
Source: Sci Rep. 2019 Dec 2;9:18019. doi: 10.1038/s41598-019-53736-8 (PMC6889138; doi:10.1038/s41598-019-53736-8)
Supplement: Supplementary file 1 — Supplementary Information [file 41598_2019_53736_MOESM1_ESM.pdf]

Molecular and structural insights into an asymmetric proteolytic complex (ClpP1P2) from *Mycobacterium smegmatis*

Jyotsna Nagpal[1], Jason J. Paxman[1], Jessica E. Zammit[1], Adam A. Thomas[1], Kaye N. Truscott[1], Begoña Heras[1,*] and David A. Dougan[1,*]

[1]Department of Biochemistry, La Trobe Institute for Molecular Science, La Trobe

*Correspondence to: David A. Dougan, Tel: +61 3 9479 3276; E-mail: d.dougan@latrobe.edu.au. or Begoña Heras, Tel: +61 3 9479 3185; E-mail: b.heras@latrobe.edu.au

Running Title: Regulation of an asymmetric ClpP1P2 complex

Keywords: ClpP, processing, regulation, degradation, AAA+ unfoldase

Supplementary Material and Methods

N-terminal sequencing (Edman degradation)

Protein fragments were separated by SDS-PAGE, transferred to a PVDF membrane in CAPS buffer (10 mM 3-[cyclohexylamino]-1 propane sulfonic acid (CAPS) pH 11, 10% (v/v) methanol), then stained using CBB. Following staining, the appropriate band (~10-20 pmol) was excised from the membrane and 5 cycles of Edman degradation performed by the Australian Proteome Analysis Facility (APAF), using an Applied Biosystems 494 Procise Sequencing system.

Antibodies (and detection)

Polyclonal antibodies against ^{Msm}ClpP1 *and* ^{Msm}ClpP2 were raised in rabbit using peptides (CSASVNGEGPGAGLDK and CTVLQYRKLSAQTS, respectively) conjugated to KHL as the antigen. Following western transfer using a semi-dry system, the proteins of interest were immunodecorated with either α-ClpP1 or α-ClpP2 antisera (1:1000) followed by a goat-α-rabbit horseradish peroxidase-coupled secondary antibody and detected using "*in house*" enhanced chemiluminescence reagents. Imaging was performed using ChemiGenius2 Bio-Imaging System (SynGene) and the digital images captured using GeneSnap software (SynGene).

Supplementary Figure legends

Supplementary Figure S1 Isolation of the ^{Msm}ClpP1P2 complex *ex vivo*.
a. Cartoon representation of ^{Msm}ClpP1 and ^{Msm}ClpP2-H_{10} encoded by synthetic operon. **b-c**. The co-expression of ^{Msm}ClpP1 and ^{Msm}ClpP2-H_{10} in *E. coli* was monitored before induction (t_0, lane 1) and four hours (t_4) post-induction (lane 2) the protein complex was purified by IMAC (lane 3). Proteins were then separated by 15% Tris-Glycine SDS-PAGE, transferred to PVDF membrane and probed with specific antisera against ClpP1 (**b**) or ClpP2 (**c**). To ensure the specificity of each antisera, purified ClpP1$_{H10}$ (lane 5) and ClpP2$_{H10}$ (lane 4) were also included.

Supplementary Figure S2 Propeptide processing of ^{Mtb}ClpP1P2 requires the addition of an activator (z-LL).

a. Cartoon representation of ^{Mtb}ClpP1 and ^{Mtb}ClpP2 indicating the position of the propeptide (grey) and the catalytic Ser residues (residue 98 for ^{Mtb}ClpP1 and 110 for ^{Mtb}ClpP2). **b.** *In vitro* processing of ^{Mtb}ClpP1P2 in the presence (lanes 1 - 5) or absence of z-LL (lanes 6-10). Wild type ^{Mtb}ClpP2 is processed (into (p) ^{Mtb}ClpP2; p^{Mtb}ClpP2) before processing of wild type ^{Mtb}ClpP1 (into (p) ^{Mtb}ClpP1; p^{Mtb}ClpP1). No processing was observed in the absence of z-LL. Following processing proteins were separated by SDS-PAGE and visualised by staining with Coomassie Brilliant Blue (CBB).

Supplementary Figure S3 The active site Ser of ^{Msm}ClpP1 (and not ^{Msm}ClpP2) is essential for peptide degradation.

The degradation of **(a)** AAF-amc **(b)** N-suc-LY-amc and **(c)** GFP-ssrA was monitored by fluorescence at (**a-b**) 460 nm (ex = 380 nm) or (**c**) 500 nm (ex = 410 nm). The rate of peptide degradation (**a-b**) was determined in the absence (lanes 1, 3 and 5) or presence (lanes 2, 4 and 6) of z-LL. (**c**) The ^{Ec}DNClpX-mediated turnover of GFP-ssrA was monitored in the absence of any addition (green circles) or in the presence of either a wild type ClpP1P2 complex (black circles), a ClpP1P2 complex bearing inactive ClpP1 (red squares) or a ClpP1P2 complex bearing inactive ClpP2 (blue triangles). Degradation rates were determined from three independent experiments (n = 3), Error bars represent SEM.

Supplementary Figure S4 Oligomeric analysis of ClpP1 and ClpP2.

a. Wild type and mutant ClpP1 were analysed by sedimentation velocity analytical ultracentrifuction (SV-AUC) analysis (left panel) and Native PAGE (right panel). The continuous standardised sedimentation distribution [*c*(*s*)] shows wild type ClpP1 (filled circles) in comparison to ClpP1^{R168A} (red circles), ClpP1^{Y60A} (green triangles), ClpP1$^{Y88'}$ (black triangles) and ClpP1^{Y110A} (blue squares) each at 0.5 mg ml^{-1} exist primarily as 5.4 $s_{20,w}$ species corresponding to a heptamer of ~ 160 kDa. Native PAGE analysis demonstrates that with the exception of ClpP1^{Y60A} (lane 3), wild type ClpP1 (lane 2), ClpP1$^{Y88'}$ (lane 4) and ClpP1^{Y110A} (lane 5) each assemble into 14-mers. **b.** Wild type and mutant ClpP2 were analysed by SV-AUC analysis. The continuous standardized sedimentation distribution [*c*(*s*)] shows wild type ClpP2 in comparison to

ClpP2^{R189A} (red circles) and ClpP2^{dbl} (blue squares) at 0.5 mg ml^{-1} exist primarily as 5 $s_{20,w}$ species corresponding to a heptamer of ~ 170 kDa.

Supplementary Figure S5 Mutation of the Hp in *^{Msm}*ClpP1 and *^{Msm}*ClpP2 modulate peptide degradation rates.

The rate of degradation of AAF-amc (**a** and **c**) and N-suc-LY-amc (**b** and **d**) is modulated by specific Hp mutations in either ^{Msm}ClpP1 (**a – b**) or ^{Msm}ClpP2 (**c – d**). Degradation rates are determined from three independent experiments. Error bars represent SEM.

Supplementary Figure S6 Single point mutations in the Hp in ^{Msm}ClpP2 (but not ^{Msm}ClpP1) inhibit the ^{Ec}DNClpX-mediated degradation of GFP-ssrA.

The ^{Ec}DNClpX-mediated degradation of GFP-ssrA was monitored by fluorescence in the absence of further additions (green triangles) in the presence of wildtype ClpP1P2 (black diamonds), ClpP1$_{Y60A}$P2 (red squares), ClpP1$_{Y88I}$P2 (pink circles), ClpP1P2$_{Y79A}$ (blue circles) or ClpP1P2$_{L129A}$ (blue squares). Degradation rates were determined from three independent experiments. Error bars represent SEM.

Supplementary Figure S7 Alignment of ClpP loop region between Sensor 1 and Box VII.

Protein sequence alignment of the sensor 1 to Box VII region of ClpX (upper panel) and ClpA/ClpC (lower panel). Upper panel contains protein sequences of *Escherichia coli* ClpX (P0A6H1), *Salmonella typhi* ClpX (Q8Z8V1), *Bacillus subtilis* ClpX (P50866), *Mycobacterium tuberculosis* ClpX (P9WPB9) and *Mycobacterium smegmatis* ClpX (A0R196). Lower panel contains protein sequences of *Escherichia coli* ClpA (P0ABH9), *Caulobacter vibrioides* ClpA (Q9A5H9), *Bacillus subtilis* ClpC (P37571), *Mycobacterium tuberculosis* ClpC1 (P9WPC9) and *Mycobacterium smegmatis* ClpC1 (A0R574).

Supplementary Figure S8 Alignment of Hp residues Y60, Y88 and Y110 in ^{Msm}ClpP1 homologs.

Protein sequence alignment of ClpP (residues 59 – 156, ^{Ec}ClpP numbering). Protein sequences of *Escherichia coli* ClpP (P0A6G7), *Mycobacterium tuberculosis* ClpP1 (MRA_2487), *Mycobacterium tuberculosis* ClpP2 (MRA_2486), *Mycobacterium smegmatis* ClpP1 (MSMEG_4673), *Mycobacterium smegmatis* ClpP2

(MSMEG_4672). *Neisseria meningitides* ClpP (X5F7H5_NEIME), *Helicobater pylori J99* ClpP (CLPP_HELPJ) and *Campylobacter jejuni* ClpP (P54413). Y76, Y104 and F126 (^{Ec}ClpP numbering), equivalent to Y60, Y80 and Y110 (^{Msm}ClpP1 numbering) are in red.

Supplementary Figure S9 Comparison of side-wall channels in ^{Msm}ClpP1 tetradecamer and ^{Mtb}ClpP1P2 tetradecamer.

Surface representation of (**a**) ^{Msm}ClpP1 tetradecamer (slate blue) and (**b**) ^{Mtb}ClpP1P2 tetradecamer (ClpP1 is shown in light blue; ClpP2 is shown in orange) reveal solvent facing side-wall channels. In ^{Msm}ClpP1, these breaches are located in the equatorial interface of the ClpP1P1 tetradecamer adjacent to the catalytic triad. In ^{Mtb}ClpP1P2 (PDB: 4U0G), the small solvent channels localise between ClpP1 subunits in the substrate binding site above the equatorial interface.

Supplementary Figure S10 Deletion of ClpP1 CTE activated peptidase activity but did not alter ATPase-mediated substrate delivery.

The rate of degradation of (**a**) AAF-amc (**b**) GFP-ssrA and (**c**) FITC-casein was monitored by fluorescence in the presence of wild type ClpP1P2 (black bars), $ClpP1_{DCTE}P2$ (white bars), $ClpP1P2_{dbl}$ (light grey bars) and $ClpP1_{DCTE}P2_{dbl}$ (dark grey bars). Degradation rates were determined from three independent experiments. Error bars represent SEM.

Table S1: Oligonucleotide primers used in this study

Primer	Gene	DNA sequence (5'-> 3')	Primer features
nde_msP1	MSMEG_4673 (*clpP1*)	CTGATGCATATGACTGA CATGCGTGGCACCGGG C	Nde I restriction site for cloning into pET10C
msP1-not	MSMEG_4673	GTCAGCGCGGCCGCTT TGTCTAGTCCTGCTCC CGGTCC	Not I restriction site for cloning into pET10C
S95A_1	MSMEG_4673	CATGGGCATGGCGGCC GCGATGGGTGAGTTCC TGC	Introduce S95A mutation
S95A_1	MSMEG_4673	CACCCATCGCGGCCGC CATGCCCATGGCGTAG GTG	Introduce S95A mutation
R168A_1	MSMEG_4673	CGATCGCTGGTTCACC GCACAGGAAGC	Introduce R168A mutation, introduces Pvu I restriction site
R168A_2	MSMEG_4673	GCGTCCGAATCGGCCT CGATGCGCTCG	Introduce R168A mutation, introduces Pvu I restriction site
73-Y60A-fwd	MSMEG_4673	GATATCCACCTGGCCA TCAACTCGCCCGGCGG C	Introduce Y60A mutation, introduces Eco RV restriction site
73-Y60Arev	MSMEG_4673	CTTGGTCGGATCTTCC GCCGACAGCAACAGGA TC	Introduce Y60A mutation,
73-Y110A-fwd	MSMEG_4673	CAAGCGCGCCGCCCTG CCCCATGCGCGC	Introduce Y110A mutation,
73-Y110-rev	MSMEG_4673	CCCTTGGTGCCCGCAG CGAGCAGGAACTC	Introduce Y110A mutation, removes Sac II restriction site
PvuI-Y88I-fwd	MSMEG_4673	TCGCCACGATCGCCAT GGGCATGGCCG	Introduce Y88I mutation,
PvuI-Y88I-rev	MSMEG_4673	CCATGGCGATCGTGGC GATGTCGCACGGC	Introduce Y88I mutation, introduces Pvu I restriction site

Sac2_4673	MSMEG_4673	CAGCCTCCGCGGTGGA ACTGACATGCGTGGCA CCGGGC	Amplification of *clpP1* Sac II restriction site for cloning into pHUE
dC_4673	MSMEG_4673	GCATCAGGATCCAAGC TTAGCTGGTGATGATGT GATCGACG	Amplification of *clpP1_{DCTE}* Hind III restriction site for cloning into pHUE
nde-ms72	MSMEG_4672 (*clpP2*)	CTGATGCATATGAGCA ACATTCATCCGTCACTG GAC	Nde I restriction site for cloning into pET10C
ms72-not	MSMEG_4672	ATCAGCGCGGCCGCCG ACGTCTGCGCAGAGAG CTTGC	Not I restriction site for cloning into pET10C
sac2-RYIL	MSMEG_4672	GCGCCTCCGCGGTGGA CGCTACATCCTGCCGT CCTTC	Sac II restriction site for cloning into pHUE
3'ms72-hind3	MSMEG_4672	AGCAGCAAGCTTACGA CGTCTGCGCAGAGAGC	Hind III restriction site for cloning into pHUE
S114A_1	MSMEG_4672	GCCAGGCCGCGGCGG CCGCGGCCGTGCTGCT GGC	Introduce S114A mutation
S114A_2	MSMEG_4672	CGGCCGCGGCCGCCG CGGCCTGGCCGAGGC ACAC	Introduce S114A mutation
R189_1	MSMEG_4672	GACAAGATCCTGACGG CCGAAGAGGCCAAGGA G	Introduce R189A mutation
R189_2	MSMEG_4672	GGCGTCGGTGTCCTTG CGGATCTGCGCCGGAT C	Introduce R189A mutation
72-Y62A-fwd	MSMEG_4672	GACATCACCATGGCCA TCAACTCGCCCGGTGG C	Introduce Y79A mutation, introduces Nco I restriction site
72-Y62-rev	MSMEG_4672	GCGGTCGGGATCCAGC GACTCCAGCACCAGCA G	Introduce Y79A mutation,

72-L111A-fwd	MSMEG_4672	GCACGCCCGGGAAGC GTGCGGCGCTGCCGAA CG	Introduce L129A mutation,
72-L111-rev	MSMEG_4672	CCGCCGCCAGCAGCAC GGCCGCTGCCGACGC	Introduce L129A mutation,
Nde-Mtb1512	^{Mtb}clpP1	CTGATGCATATGAGCC AAGTGACTGACATGCG	Nde I restriction site for cloning into pET10C
Mtb1512-not	^{Mtb}clpP1	CTAGCAGCGGCCGCCT GTGCTTCTCCATTGAC GTGGG	Not I restriction site for cloning into pET10C
Nde-Mtb1513	^{Mtb}*clpP*2	CATGTCCATATGAATTC CCAAAATTCTCAGATCC	Nde I restriction site for cloning into pET10C
Mtb1513-not	^{Mtb}*clpP*2	TCGTCAGCGGCCGCGG TTTGCGCGGAGAGC	Not I restriction site for cloning into pET10C
Msm-ssrA-1	^{Msm}ssrA	GGCCGCCGATTCCAAT CAGCGCGACTACGCCC TCGCTGCCTA	Anneal with Msm-ssrA-2, ligated between Not I - Hind III restriction sites in pDD173 (Dougan et al., 2002)
Msm-ssrA-2	^{Msm}ssrA	AGCTTAGGCAGCGAGG GCGTAGTCGCGCTGAT TGGAATCGGC	Anneal with Msm-ssrA-1, ligated between Not I-Hind III restriction sites in pDD173 (Dougan et al., 2002)

[1] restriction sites for cloning and/or screening are underlined

Table S2: Plasmids used in this study

Plasmid Name	Description		Plasmid features, source
	gene	Parental plasmid	
pDT2535	*clpP1* (MSMEG_4673)	pET10C	Amplified *clpP1* using nde_msP1and msP1-not, digested with *Nde* I and *Not* I and cloned into pET10C
pDT2517	*clpP2* (MSMEG_4672)	pET10C	Amplified *clpP2* using nde_msP2and msP2-not, digested with *Nde* I and *Not* I and cloned into pET10C
pDT2723	clpP1-S95A	pET10C	Quick change mutagenesis using pDT2535 and primers S95A_1 and S95A_2
pDT2829	p-clpP2	pHUE	Amplified “processed” *clpP2 (p-clpP2)* using sac2-RYIL and 3’ms72-hind3 digested with *Sac* II and *Hind* III and cloned into pHUE
pDT2842	p-clpP2-S114A	pHUE	Quick change mutagenesis using pDT2829 and primers S114A_1 and S114A_2
pDT2872	p-clpP2-Y79A	pHUE	Quick change mutagenesis using pDT2829 and primers 72-Y62A-fwd and 72-Y62A-rev
pDT2873	p-clpP2-L129A	pHUE	Quick change mutagenesis using pDT2829 and primers 72-L111A-fwd and 72-L111A-rev
pDT2889	clpP1-Y60A	pET10C	Quick change mutagenesis using pDT2535 and primers 73-Y60A-fwd and 73-Y60A-rev
pDT2891	clpP1-Y110A	pET10C	Quick change mutagenesis using pDT2535 and primers 73-Y110A-fwd and 73-Y110A-rev
pDT3028	clpP1-DCTE	pHUE	Amplified *clpP1* using Nde_rssB and rssB_stop_xho, digested with *Nde* I and *Xho* I and cloned into pETduet-1
pDT3039	p-clpP2$_{dbl}$ = Y79A/L129A	pHUE	Quick change mutagenesis using pDT2872 and primers 72-L111A-fwd and 72-L111A-rev
pDT3082	p-clpP2-R189A	pHUE	Quick change mutagenesis using pDT2829 and primers R189_1 and R189_2

pDT3128	clpP1-R168A	pET10C	Quick change mutagenesis using pDT2535 and primers R168A_1 and R168A_2
pDT3232	clpP1-Y88I	pET10C	Quick change mutagenesis using pDT2535 and primers PvuI-Y88I-fwd and PvuI-Y88I-rev

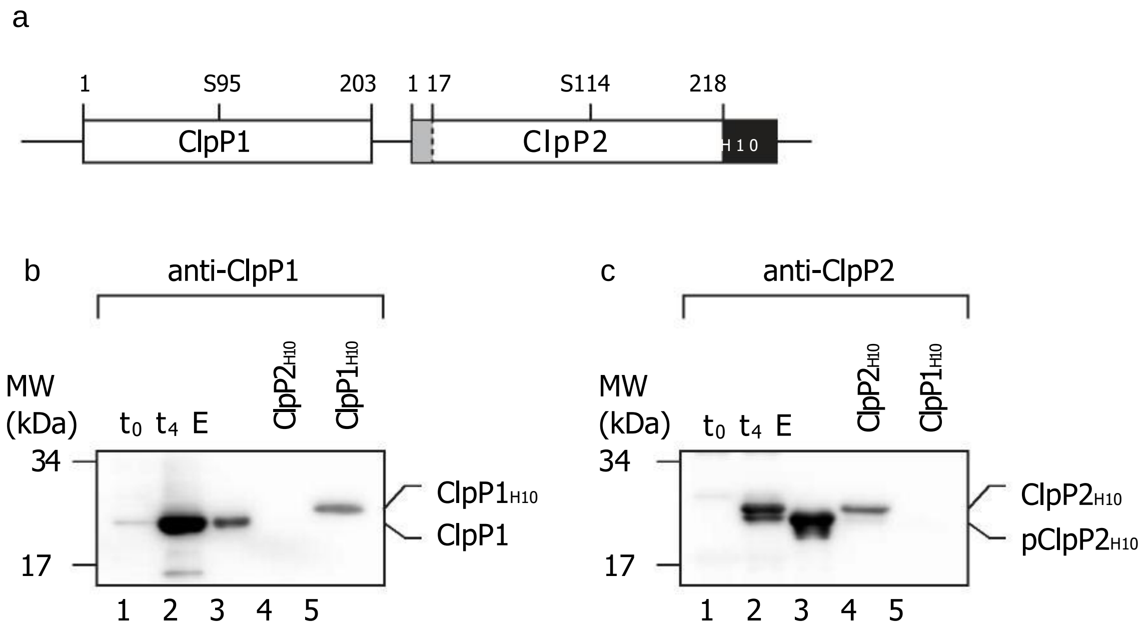

Nagpal et al., Supp Fig S1

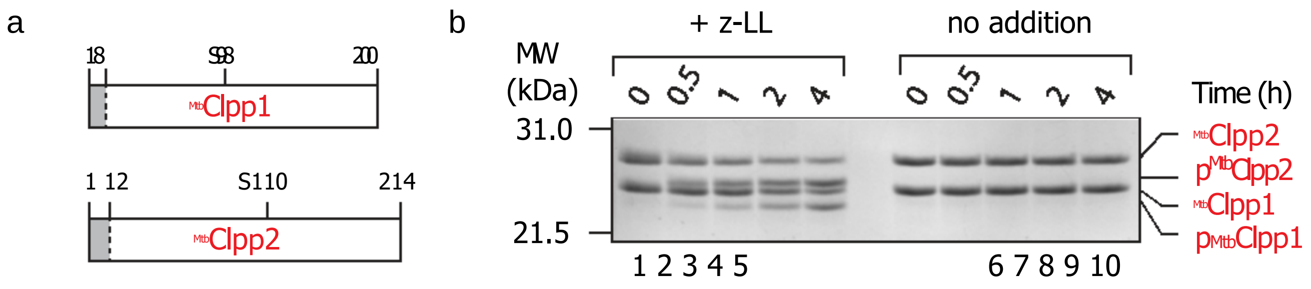

Nagpal et al., Fig S2

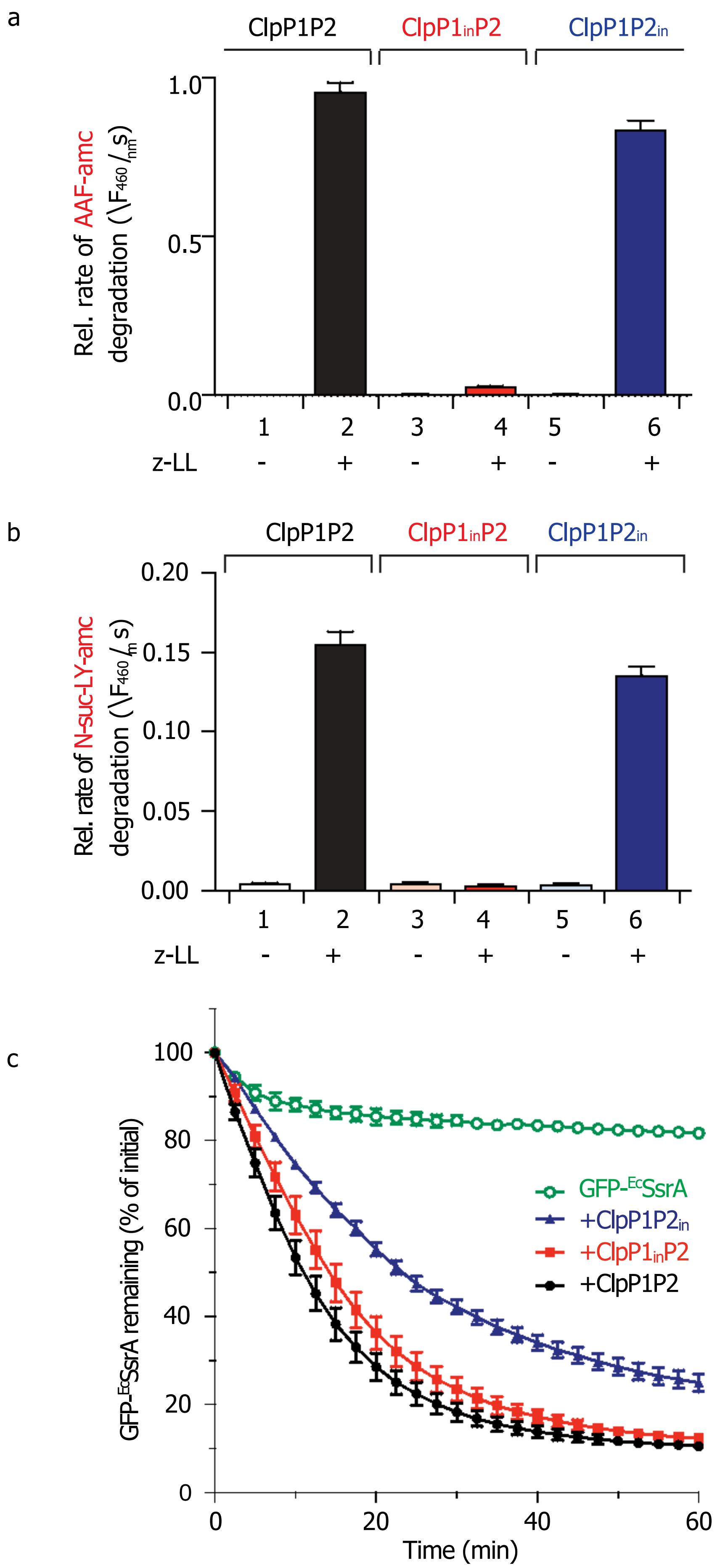

Nagpal Supp Fig S3

a

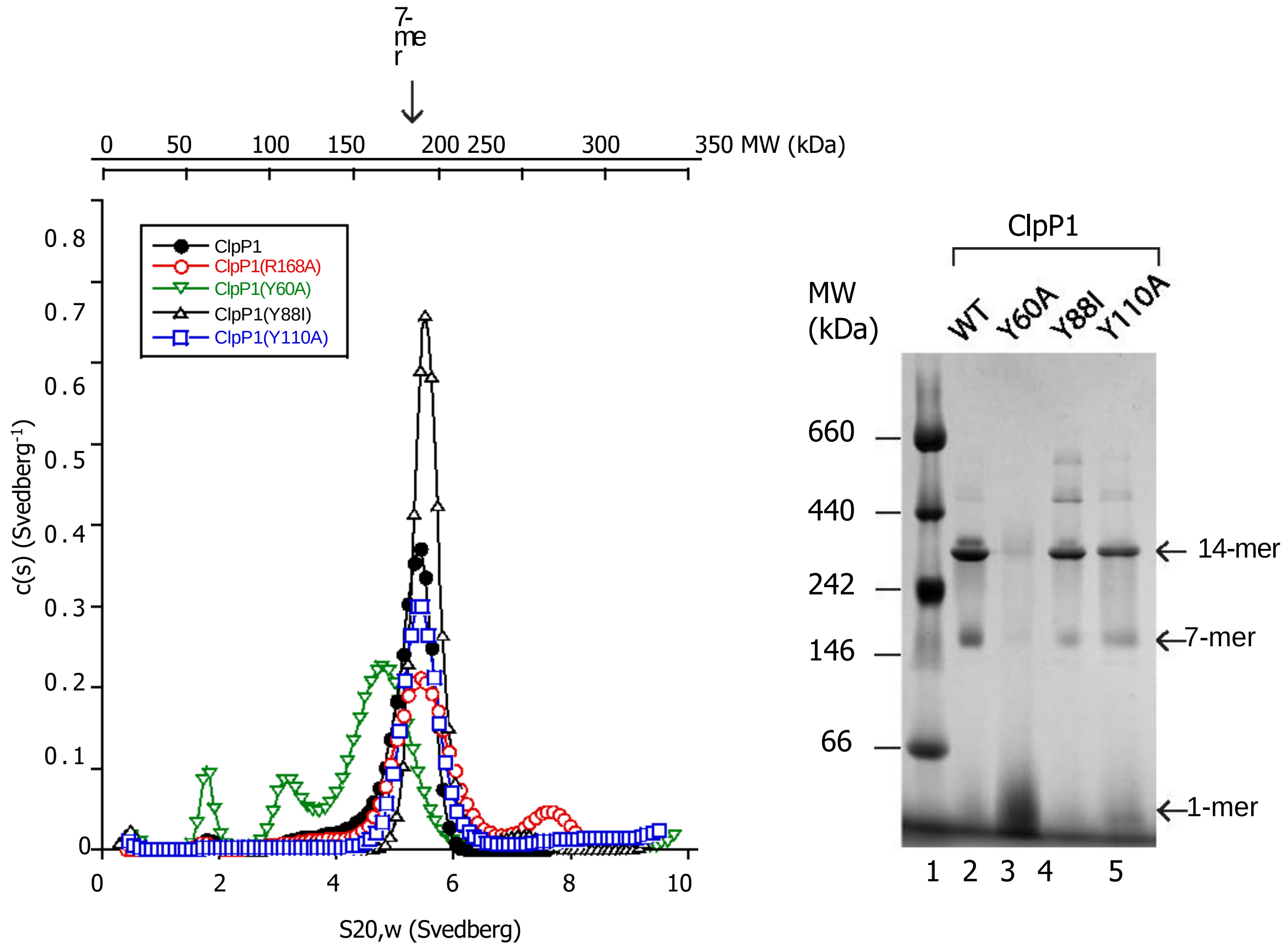

b

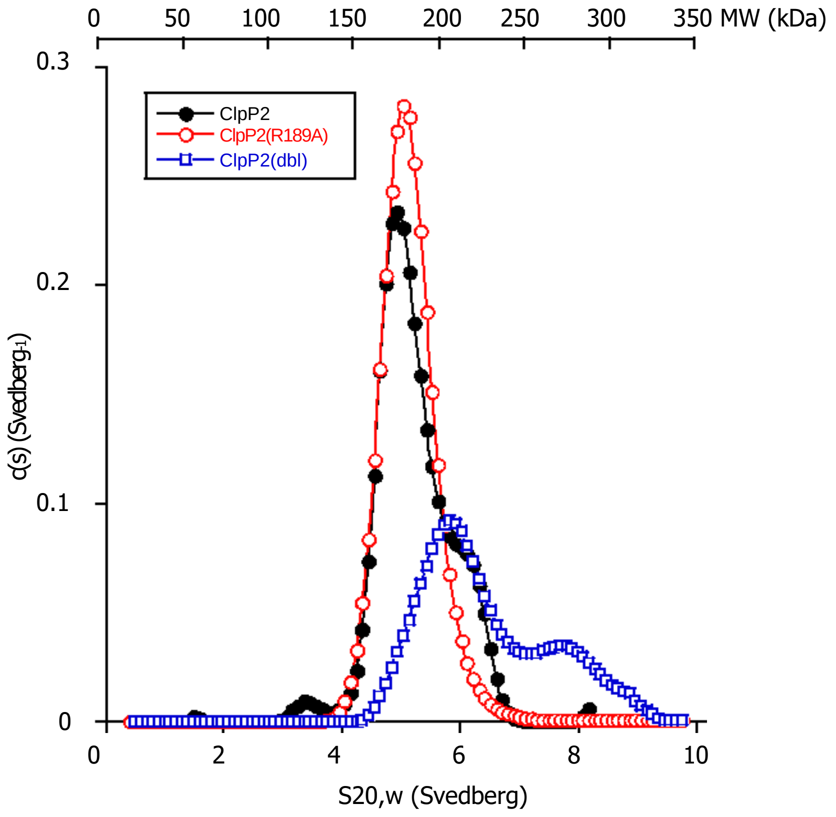

Nagpal et al., Supp Fig S4

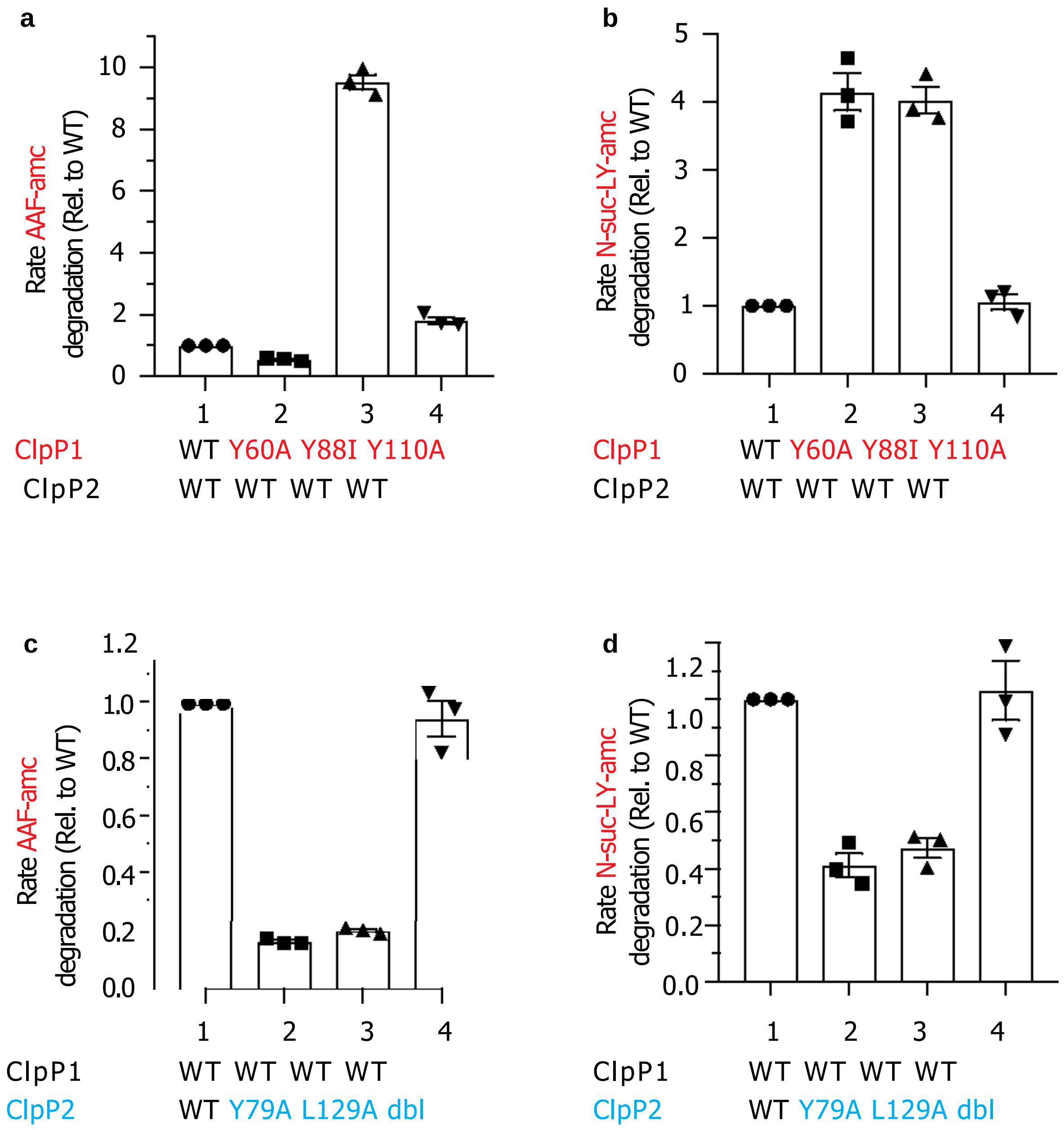

Nagpal et al., Supp Fig S5

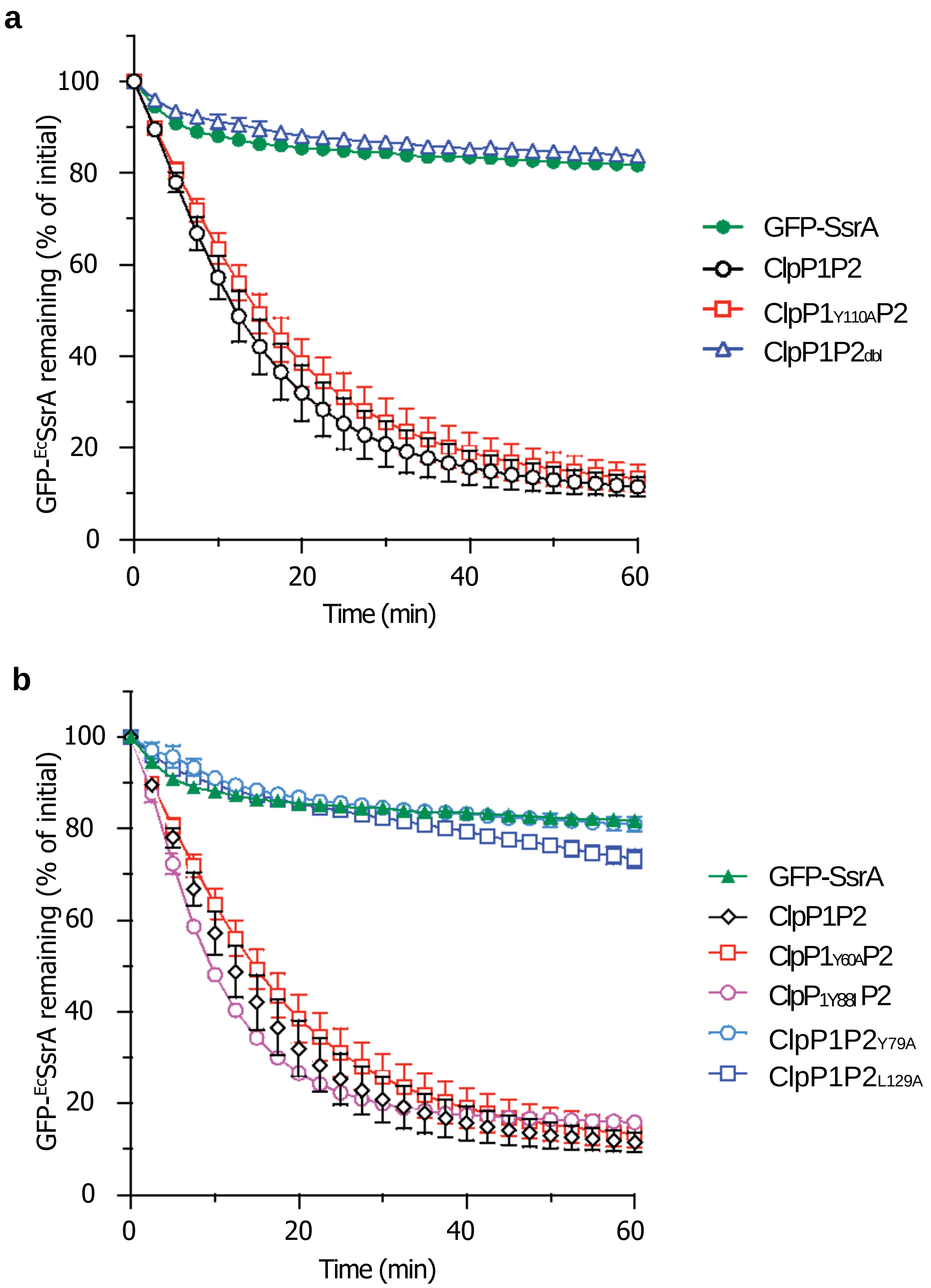

Nagpal et al., Supp Fig S6

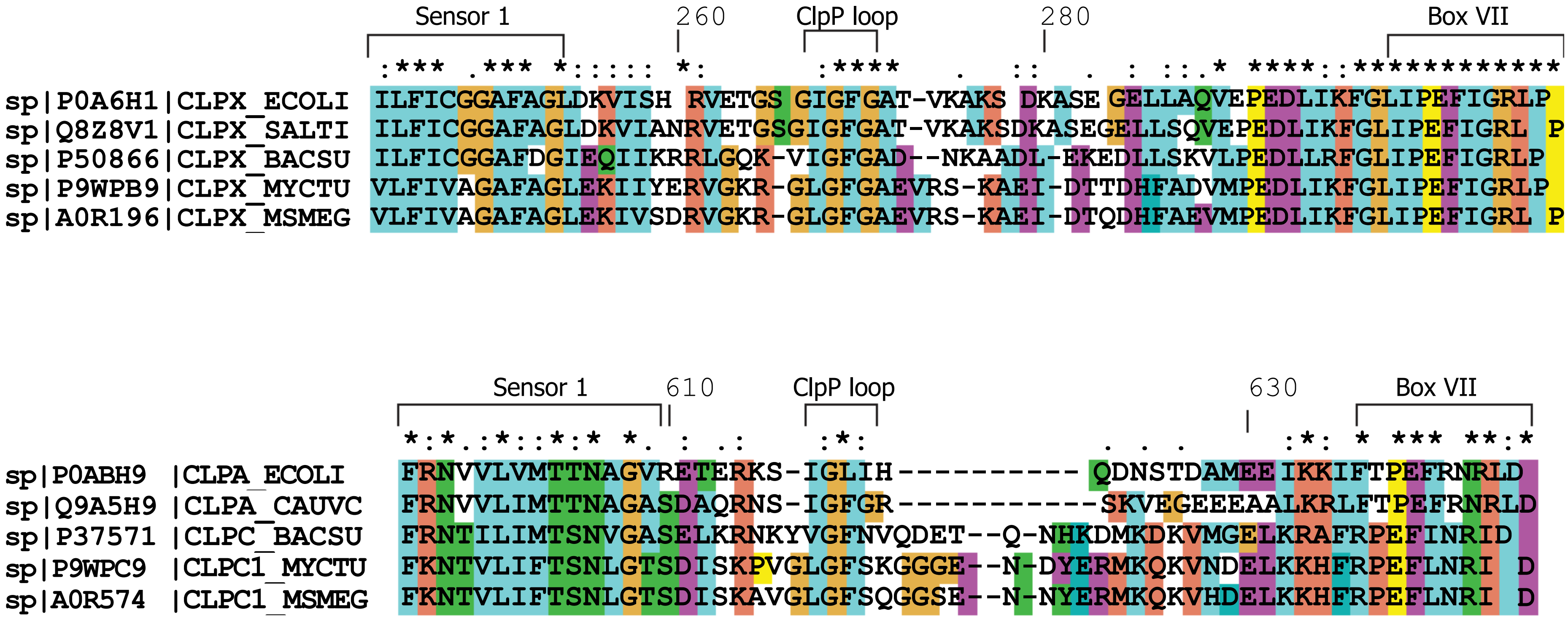

Nagpal et al., Supp Fig S7

E. coli ClpP numbering

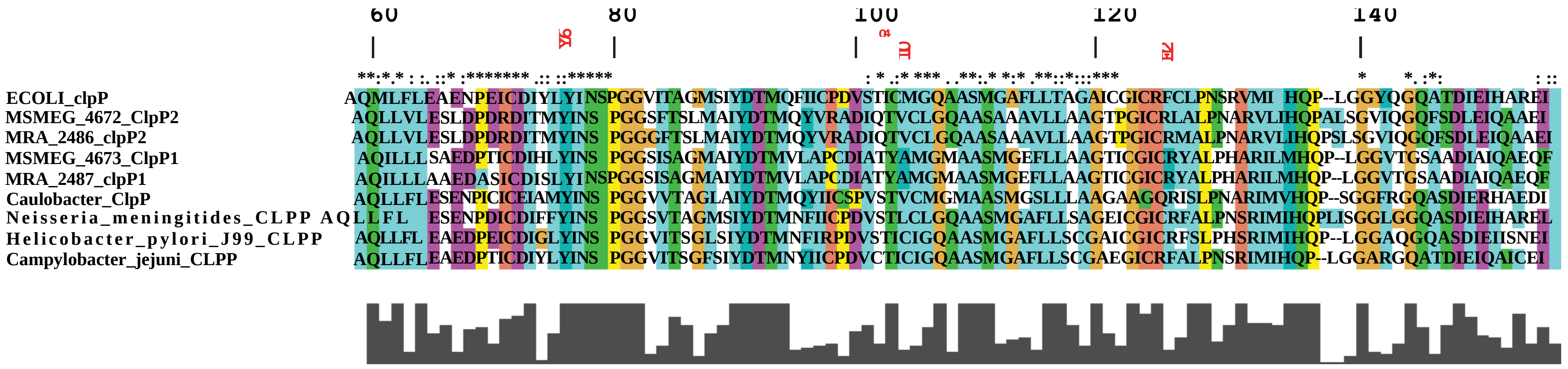

Nagpal et al., Supp Fig S8

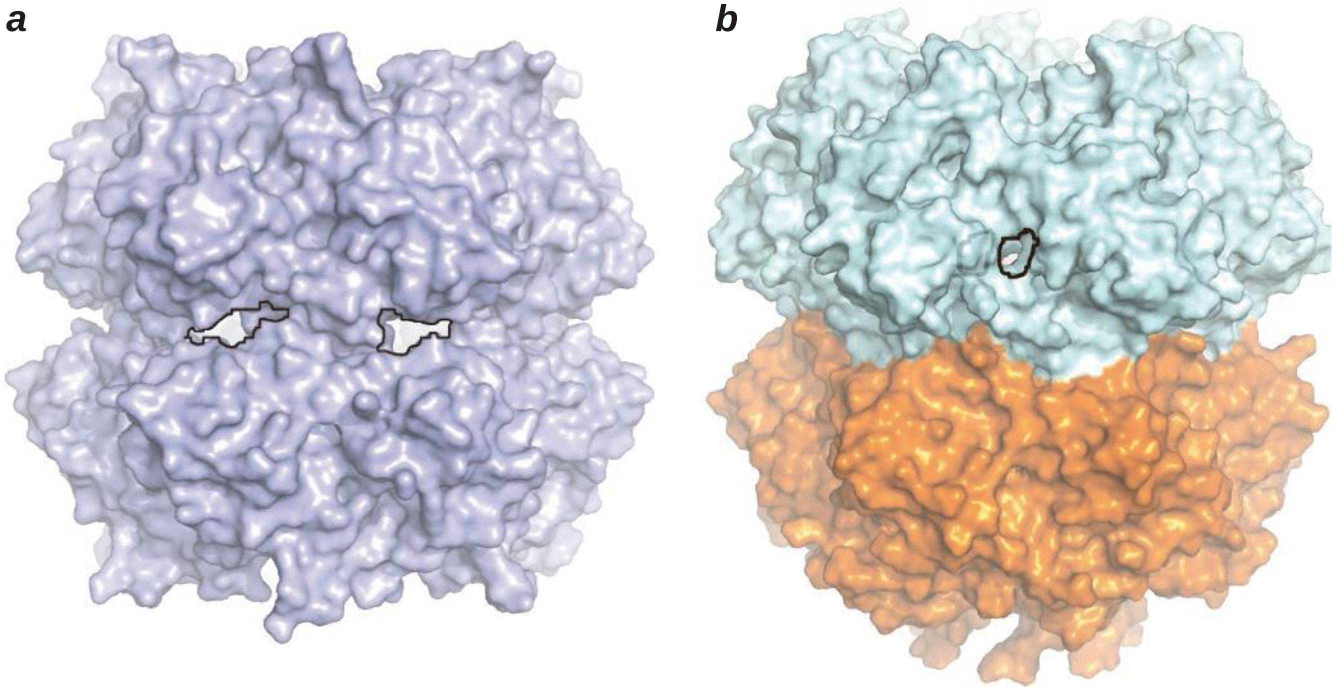

^{Msm}ClpP1 ^{Mtb}ClpP1P2

Nagpal et al., Supp Fig S9

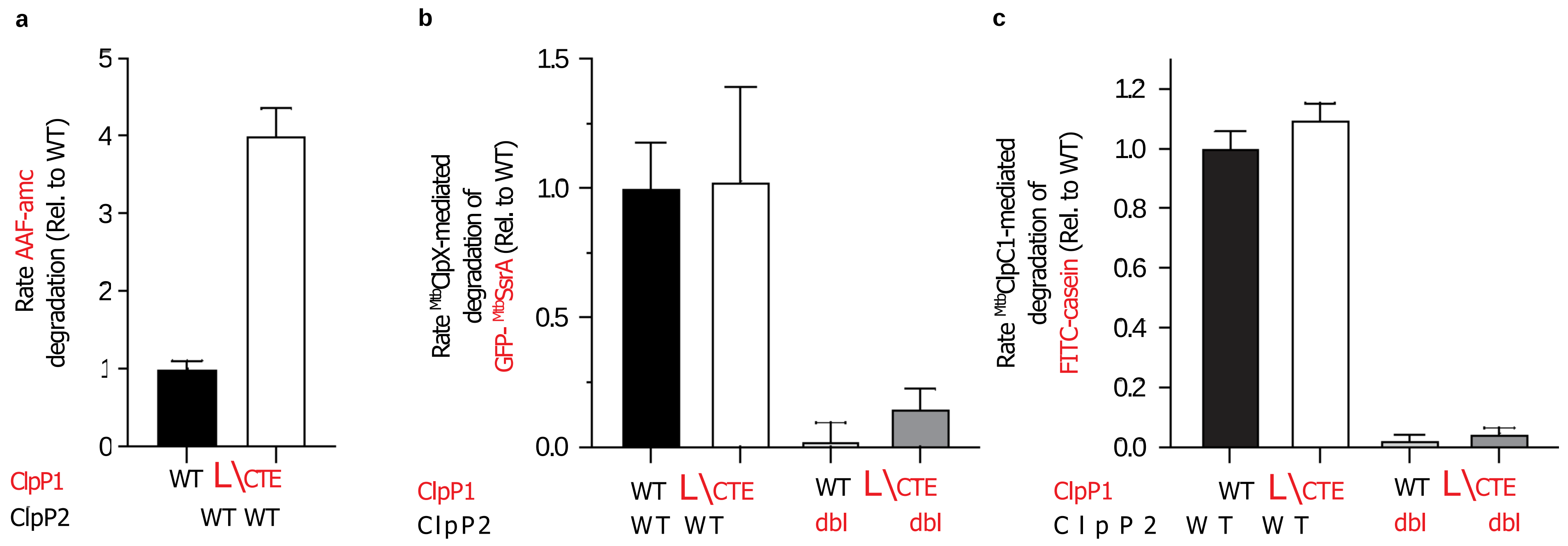

Nagpal et al., Supp Fig S10
